# Insulin Treatment May Increase Adverse Outcomes in Patients With COVID-19 and Diabetes: A Systematic Review and Meta-Analysis

**DOI:** 10.3389/fendo.2021.696087

**Published:** 2021-07-22

**Authors:** Yan Yang, Zixin Cai, Jingjing Zhang

**Affiliations:** National Clinical Research Center for Metabolic Diseases, Metabolic Syndrome Research Center, Key Laboratory of Diabetes Immunology, Ministry of Education, and Department of Metabolism and Endocrinology, The Second Xiangya Hospital of Central South University, Changsha, China

**Keywords:** insulin treatment, COVID-19, adverse outcomes, diabetes, mortality, complications

## Abstract

**Background and Objective:**

Recently, insulin treatment has been found to be associated with increased mortality and other adverse outcomes in patients with coronavirus disease 2019 (COVID-19) and diabetes, but the results remain unclear and controversial, therefore, we conducted this meta-analysis.

**Methods:**

Four databases, namely, PubMed, Web of Science, EMBASE and the Cochrane Library, were used to identify all studies concerning insulin treatment and the adverse effects of COVID-19, including mortality, incidence of severe/critical complications, in-hospital admission and hospitalization time. To assess publication bias, funnel plots, Begg’s tests and Egger’s tests were used. The odds ratios (ORs) with 95% confidence intervals (CIs) were used to access the effect of insulin therapy on mortality, severe/critical complications and in-hospital admission. The association between insulin treatment and hospitalization time was calculated by the standardized mean difference (SMD) with 95% CIs.

**Results:**

Eighteen articles, involving a total of 12277 patients with COVID-19 and diabetes were included. Insulin treatment was significantly associated with an increased risk of mortality (OR=2.10; 95% CI, 1.51-2.93) and incidence of severe/critical COVID-19 complications (OR=2.56; 95% CI, 1.18-5.55). Moreover, insulin therapy may increase in-hospital admission in patients with COVID-19 and diabetes (OR=1.31; 95% CI, 1.06-1.61). However, there was no significant difference in the hospitalization time according to insulin treatment (SMD=0.21 95% CI, -0.02-0.45).

**Conclusions:**

Insulin treatment may increase mortality and severe/critical complications in patients with COVID-19 and diabetes, but more large-scale studies are needed to confirm and explore the exact mechanism.

## Introduction

Since the rapid outbreak of coronavirus disease 2019 (COVID-19) in 2019, the worldwide fight against the epidemic is still ongoing. As of January 30, 2021, the number of confirmed cases and deaths reached 114140104 and 2535520, respectively (1). The concomitant occurrence of two worldwide epidemic diseases, COVID-19 and diabetes, has introduced a series of problems. Diabetes is known as among the most serious comorbidities of COVID-19 to deteriorate disease development and outcomes. Recently, mechanisms underlying why diabetic COVID-19 patients are more prone to severe outcomes include higher glucose levels, impaired innate and adaptive immune response, impaired coagulation, obesity and hypertension (2).

Good glucose control has been demonstrated to exert a protective effect in the context of the development and outcomes of COVID-19 (3–5), and strict glucose management on admission for COVID-19 cases has been suggested regardless of diabetes status (6). The role of glycemic control strategies, such as several antidiabetic drugs including metformin and DPP-4 inhibitors in the efficacy and safety of COVID-19 and diabetes has been established (7, 8). However, whether insulin injection has some effect on the development of COVID-19 remains inconsistent. Notably, early research found that insulin treatment may be associated with adverse outcomes such as death (9) and intensive care unit (ICU) admission (10); however, evidence regarding the relationship between insulin treatment and COVID-19 remains inadequate and unconvincing. Therefore, we conducted a systematic review and meta-analysis to determine the association between insulin injection and the outcomes of COVID-19 to provide certain clinical information for these patients.

## Methods

This meta-analysis was conducted according to the Preferred Reporting Items for Systematic Reviews and Meta-Analyses (PRISMA) statement guidelines, as previously described (11).

### Article Search Strategy

We searched for eligible articles from January 25, 2021 and to February 20, 2021. The PubMed (2013-2020, 20 February), Cochrane Library (1960-2020, 20 February), EMBASE (1960-2020, 20 February) and Web of Science (1950-2020, 20 February) databases were searched in this study. Searches for all published articles related to both insulin treatment and COVID-19 were performed. The following search terms were used: “Insulin”, “Insulin treatment”, “COVID-19”, “SARS-CoV-2”, and ‘‘2019 novel coronavirus’’. The search strategy was as follows: [(insulin) OR (insulin treatment)] AND [(COVID-19) OR (SARS Cov-2) OR (coronavirus) OR (2019 novel coronavirus)]. Additional papers were identified by performing manual searches of the references of relevant articles and tracking citations to obtain more relevant studies. All articles published by February 20, 2021 with no language restrictions were included.

### Selection Criteria

Two reviewers (YY and ZC) independently reviewed all eligible studies and selected those suitable for inclusion. Disagreements were settled by reaching a consensus or with the help of a third reviewer (JZ). All articles included in this meta-analysis met the following criteria: (1) population: the subjects were patients with both COVID-19 and diabetes; (2) intervention: insulin treatment; (3) comparator/control: the control group involved non-insulin treatment, including oral antidiabetic medications, no treatment or diet control; (4) importantly, the outcomes of our study refer to the mortality and other complications of COVID-19, and the form of the outcomes must be original data of COVID-19 deaths and complications or calculated adjusted/unadjusted odds ratios (ORs) or risk ratios (RRs); and (5) study design: clinical studies. Articles were excluded if they met the following criteria: (1) articles lacking information or data necessary for the purpose of this meta-analysis and (2) articles published as letters, reviews, editorials, or conference abstracts.

### Data Extraction

All relevant articles were imported into EndNote X9 software and reviewed independently by two authors (YY and ZC). Discrepancies between the authors were resolved with the help of a third reviewer (JZ). The following information was extracted from the selected studies by the two independent investigators: author, publication year, country, study design, mean or median age, sample size, population and COVID-19 outcomes. All extracted data were then imported into Excel.

**Figure 1:**
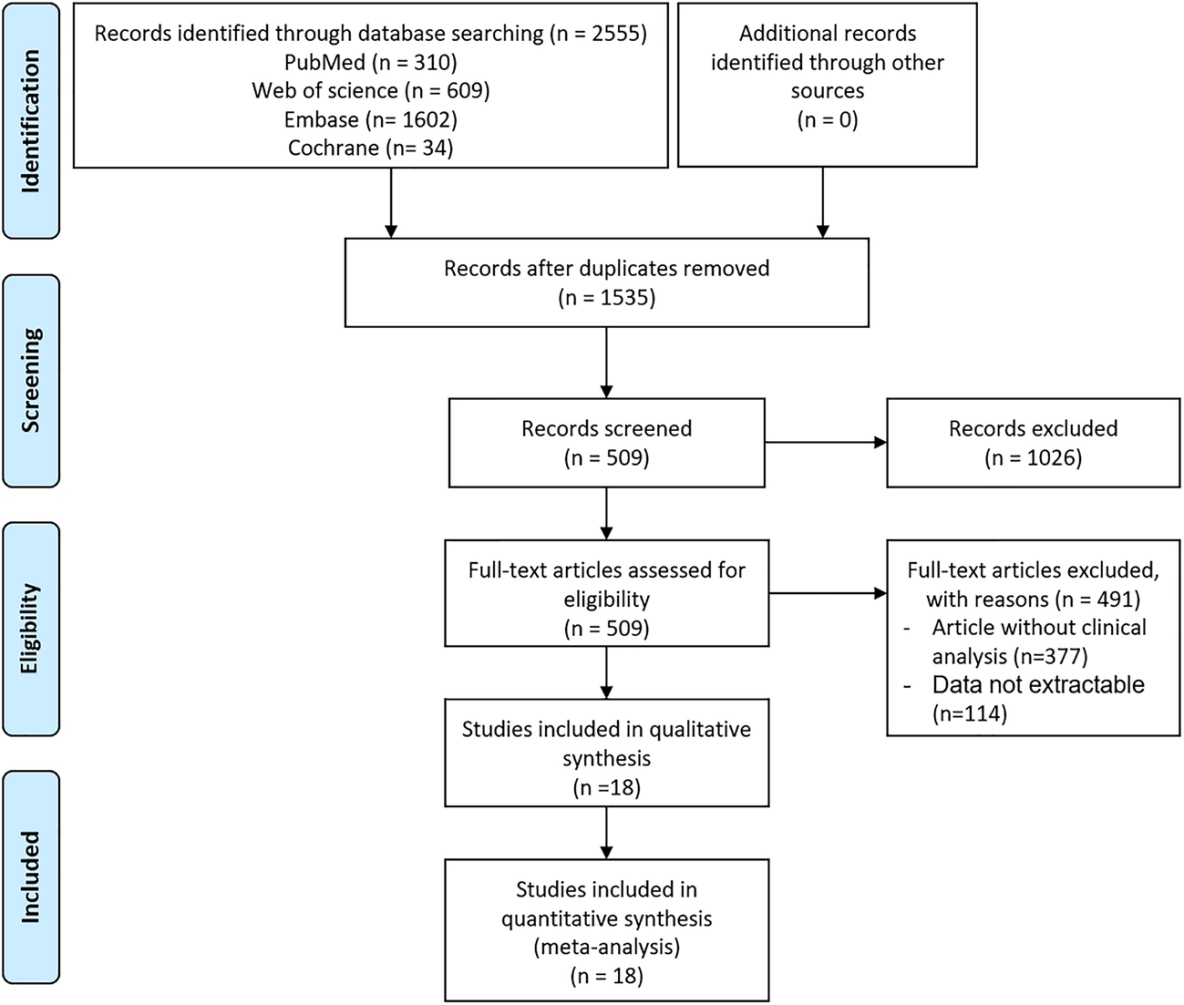
Flow diagram of the study selection process.

The severity of the disease was classified into 4 categories according to the Guidelines for the Diagnosis and Treatment of New Coronavirus Pneumonia (fifth edition) as follows (12): 1) *mild type*: patients with mild clinical symptoms and no pulmonary changes on CT imaging, 2) *common type*: patients with symptoms of fever and signs of respiratory infection, with pneumonia changes on CT imaging, 3) *severe type*: patients presenting with any of the following items: (a) respiratory distress, respiratory rate≥30/min, (b) oxygen saturation of finger ≤ 93% in resting condition, and (c) arterial partial pressure of oxygen (PaO2)/oxygen concentration (FiO2)≤300 mmHg (1 mmHg=0.133 kPa) or 4) *critical type*: patients meeting any of the following criteria: (a) respiratory failure requiring mechanical ventilation, (b) shock and (c) ICU admission requirement due to multiple organ failure. Since only 32 mild cases were available and these cases would normally not be hospitalized, these cases were removed from the analysis (**Figure 1**).

### Quality Assessment of Studies

The quality of the included studies was assessed by the Newcastle-Ottawa Scale (NOS) (13). We assessed the quality of all relevant studies based on the type of study, sample size, participant selection, representativeness of the sample, adequacy of follow-up, comparability (exposed-unexposed or case-control), and method of ascertaining the cases and controls. The possible range of NOS scores is from 0 to 9; studies that scored 4-6 represent a modest risk of bias, and those that scored <3 indicate the highest risk of bias. A study with a score of 6 or higher was defined as a high-quality study.

### Statistical Analysis

All analyses were performed using Stata (Version 13.0). The correlation between insulin treatment and mortality or incidence of complications of COVID-19 was expressed as the pooled ORs and 95% confidence interval (CI). ORs > 1 represented a direct association, and those < 1 represented an inverse association. Regarding the hospitalization time, the standardized mean difference (SMD) and 95% CI were used to analyze the results. A random-effects model was used for all results of our meta-analysis. I^2^ statistics were used to assess the degree of heterogeneity as follows: 25%, 50%, and 75% represented low, moderate, and high degrees of heterogeneity, respectively. Subgroup analyses were performed using the following variables to analyze the heterogeneity: sample size (>80 or <80), period of insulin treatment (before and after admission), type of diabetes (T1DM, T2DM and Diabetes), and control groups (with or without antidiabetic drugs). Additionally, Begg’s and Egger’s tests and funnel plots were used to detect potential publication bias, with a p-value <0.05 suggesting the presence of bias.

## Results

### Search Results and Study Characteristics

A flowchart of the study selection process is shown in **Figure 1**. After a preliminary search of the selected electronic databases, in total, 2555 studies were identified. After 1020 duplicates were eliminated, 1535 studies were selected by screening the titles and abstracts. After further excluding 1026 studies based on the titles and abstracts, 509 articles remained. Of these 509 articles, 491 were excluded for the following reasons after the full text was read: (1) not enough participant information was provided (n=377); and (2) the original data regarding insulin use were not provided (n=114). Finally, 18 articles (9, 10, 14–29) related to the use of insulin treatment and COVID-19 were included in this meta-analysis. The basic characteristics of the studies are presented in **Table 1**. Among the 18 studies included in this analysis, 2 studies were performed in France, 4 studies were performed in China, 5 studies were performed in the USA, 2 studies were performed in Italy, 1 in Russia, 1in Spain, 1 in Saudi Arabia, 1 in Iran and 1 in Iraq (**Table 1**). All included studies were published in 2020 or 2021. Additionally, as the different outcomes of COVID-19 were analyzed in our study, information regarding the association between insulin treatment and mortality is included in **Table 2**, and the outcomes of severe/critical complications or in-hospital admission are shown in **Table 3**.

**Table 1:** Description of eligible studies reporting the association between insulin treatment and the outcomes of COVID-19.

No.	Author	Country	Age	Type of study	Sample size	NOS	Population
1	Bo Yu (14)	China	66 (57–73)	a retrospective study	689	8	patients with COVID-19 and T2DM
2	Cariou (15)	France	69.8 ± 13.0	a nationwide multicentre observational study	1317	7	patients with COVID-19 and diabetes
3	Yuchen Chen (16)	China	56.0 (39.0–67.0)	a retrospective study	904	8	patients with COVID-19 and diabetes (mostly T2DM)
4	Shayan Riahi (9)	USA	66.42 ± 12.67	a retrospective cohort	166	7	patients with COVID-19 and diabetes
5	Luis M. Pérez (10)	Spain	74.9 ± 8.4	a nationwide cohort study	2666	7	patients with COVID-19 and T2DM
6	Shivani Agarwal (18)	USA	68	cohort	1126	8	patients with COVID-19 and diabetes
7	Marina V (19)	Russia	NA	a retrospective study	309	7	patients with COVID-19 and T2DM
8	Marco Mirani (20)	Italy	71 (64–78)	cohort	90	8	patients with COVID-19 and T2DM
9	Shayesteh Khalili (21)	Iran	65.7 ± 12.51	cohort	127	7	patients with COVID-19 and diabetes
10	Justin J (22)	USA	65.7 ± 15.5	a retrospective study	46	8	patients with COVID-19 and diabetes
11	Huadong Yan (23)	China	49.18 (14.16)	Case-Control Study	717	8	patients with COVID-19 and T2DM
12	Ayman A (24)	Saudi Arabia	57.6 ± 13.9	cohort	806	8	patients with COVID-19 and T2DM
13	Achille Cernigliaro (25) Italy		NA	a retrospective study	172	7	patients with COVID-19 and diabetes
14	Hussein Nafakhi (26)	Iraq	60 ± 10	a retrospective study	67	7	patients with COVID-19 and diabetes
15	Adèle Lasbleiz (27)	France	62.1 ± 14.0	cohort	2168	8	patients with COVID-19 and diabetes (mostly T2DM)
16	Michelle A (30)	USA	75.6 (10.8)	retrospective cohort study	775	8	patients with COVID-19 and T2DM
17	Grenye O’Malley (28)	USA	NA	cross-sectional study	113	7	patients with COVID-19 and T1DM
18	Ting Guo (29)	China	62.1 (42-76)	a retrospective study	19	7	patients with COVID-19 and diabetes

NA, data not available.

**Table 2:** Description of studies reporting the association between insulin treatment and the mortality of COVID-19.

No.	Study	Sample size	Period of insulin treatment	Type of diabetes	Control groups	or	ci1	ci2
1	Bo Yu 2021 (14)	689	after admission	T2DM	with or without antidiabetic drugs	5.38	2.75	10.54
2	Cariou 2020 (15)	1317	before admission	diabetes	antidiabetic drugs	1.71	1.2	2.43
3	Yuchen Chen 2020 (16)	904	before admission	diabetes	with or without antidiabetic drugs	4.461	1.223	16.266
4	Shayan Riahi 2020 (9)	166	before admission	diabetes	antidiabetic drugs	2.486	1.204	5.134
5	Luis M. Pérez 2020 (10)	2666	after admission	T2DM	antidiabetic drugs	1.45	1.11	1.88
6	Shivani Agarwal 2020 (18)	1126	before admission	diabetes	without antidiabetic drugs	2.3	1.32	4.01
7	Marina V 2020 (19)	309	before admission	T2DM	antidiabetic drugs	2.67	1.42	5.02
8	Marco Mirani 2020 (20)	90	before admission	T2DM	antidiabetic drugs	3.05	1.57	5.95
9	Shayesteh Khalili 2020 (21)	127	before admission	diabetes	antidiabetic drugs	0.242	0.061	0.967
10	Justin J 2020 (22)	46	before admission	diabetes	with or without antidiabetic drugs	11.873	2.218	63.555
11	Achille Cernigliaro 2020 (25)	172	before admission	diabetes	antidiabetic drugs	1.87	0.89	3.89
12	Michelle A 2020 (30)	775	before admission	T2DM	with or without antidiabetic drugs	1.197	0.731	1.962

**Table 3:** Description of studies reporting the association between insulin treatment and the severe/critical complications or in-hospital admission of COVID-19.

No.	Study	Sample size	Complications	Period of insulin treatment	Type of diabetes	Control groups	or	ci1	ci2
1	Bo Yu (14)	689	ICU admission	after admission	T2DM	with or without antidiabetic drugs	11.47	3.4	38.66
2	Bo Yu (14)	689	Invasive mechanical ventilation	after admission	T2DM	with or without antidiabetic drugs	6.73	3.39	13.37
3	Yuchen Chen (16)	904	Poor prognosis	before admission	Diabetes	with or without antidiabetic drugs	3.58	1.37	9.35
4	Luis M. Pérez (10)	2666	ICU admission;	after admission	T2DM	antidiabetic drugs	1.32	1.02	1.71
5	Huadong Yan (23)	717	severity	before admission	T2DM	antidiabetic drugs	2.71	1.6	5.5
6	Hussein Nafakhi (26)	67	extensive lung injury	before admission	Diabetes	antidiabetic drugs	0.4	0.3	5
7	Hussein Nafakhi (26)	67	Partial recovery with persistent symptoms	before admission	Diabetes	antidiabetic drugs	0.3	0.2	4
8	Ting Guo (29)	19	ICU admission	before admission	Diabetes	without antidiabetic drugs	115	4.115	3213.5
No.	Study	Sample size	Complications	Period of insulin treatment	Type of diabetes	Control groups	or	ci1	ci2
1	Luis M. Pérez (10)	2666	In-hospital admission	after admission	T2DM	antidiabetic drugs	1.23	0.95	1.6
2	Ayman A (24)	806	In-hospital admission	before admission	T2DM	antidiabetic drugs	1.46	1.03	2.07
3	Achille Cernigliaro (25)	172	In-hospital admission	before admission	Diabetes	antidiabetic drugs	2.13	1.01	4.49
4	Adèle Lasbleiz (27)	2168	In-hospital admission	before admission	Diabetes	antidiabetic drugs	3.8	1.9	8.1
5	Grenye O’Malley (28)	113	In-hospital admission	after admission	T1DM	without antidiabetic drugs	0.137	0.053	0.352

### Quality Assessment

NOS mainly consist of the following three aspects: sample selection, comparability of cases and controls, and exposure. All included studies had NOS scores higher than 6, indicating the high quality of our studies. The details of the risk of bias are described in **Table 1**.

### Primary Outcomes

#### Insulin Treatment and COVID-19 Mortality

**Figure 2:**
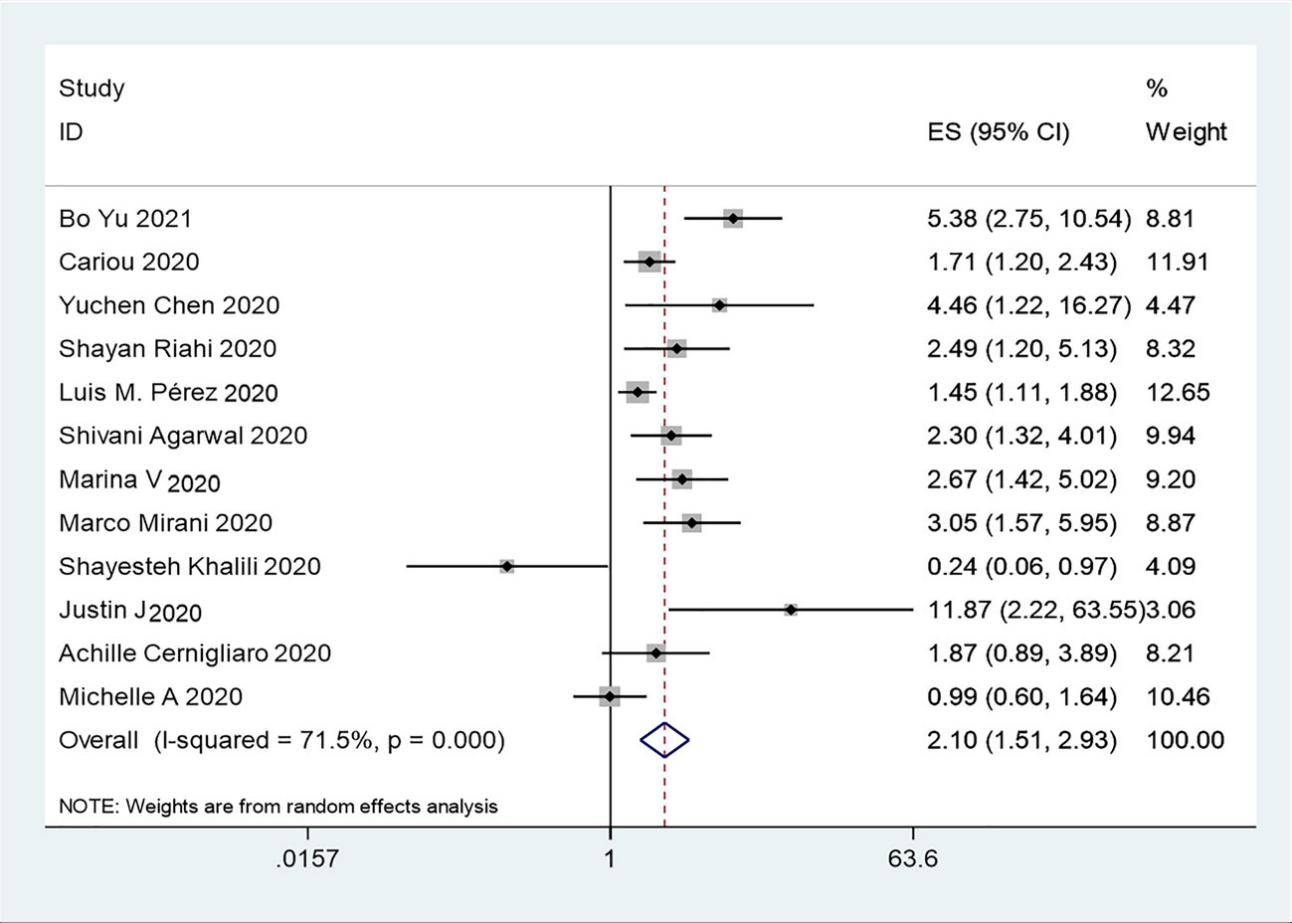
Forest plots of OR for the association between the insulin treatment and the mortality of COVID-19.

**Figure 3:**
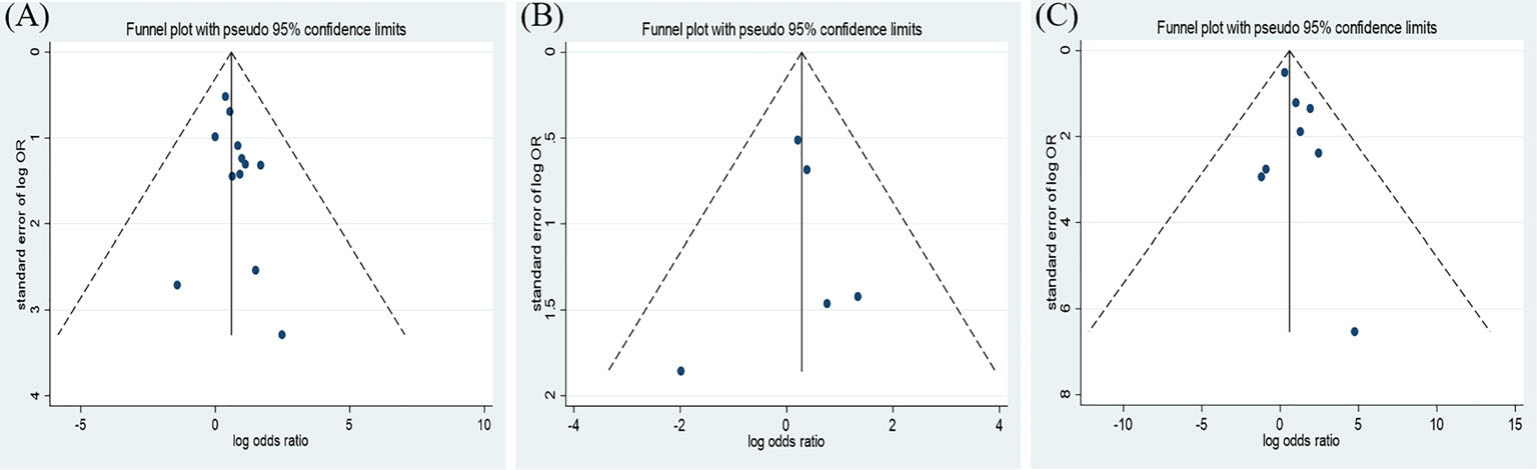
Funnel plot of the association between the insulin treatment and the mortality **(A)**, complications **(B)**, and the in-hospital admission **(C)** of COVID-19.

**Figure 4:**
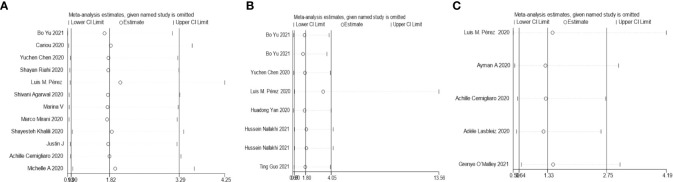
Sensitivity analysis for the effect insulin treatment on the mortality **(A)**, complications **(B)**, and the in-hospital admission **(C)** of COVID-19.

The results of this meta-analysis of the use of insulin and COVID-19 mortality are shown in **Figure 2**. In general, the use of insulin is associated with increased mortality due to COVID-19 (OR=2.10 95% CI, 1.51-2.93); high heterogeneity was observed (I^2^ statistic=71.5%, p<0.001) (**Figure 2**). The results of Egger’s (p = 0.2) and Begg’s tests (p = 0.193) and inspection of the funnel plots showed that there was no publication bias among the studies (**Figure 3A**). A sensitivity analysis was conducted by omitting one study at a time and showed that the results were stable (**Figure 4A**).

#### Insulin Treatment and COVID-19 Severe/Critical Complications

**Figure 5:**
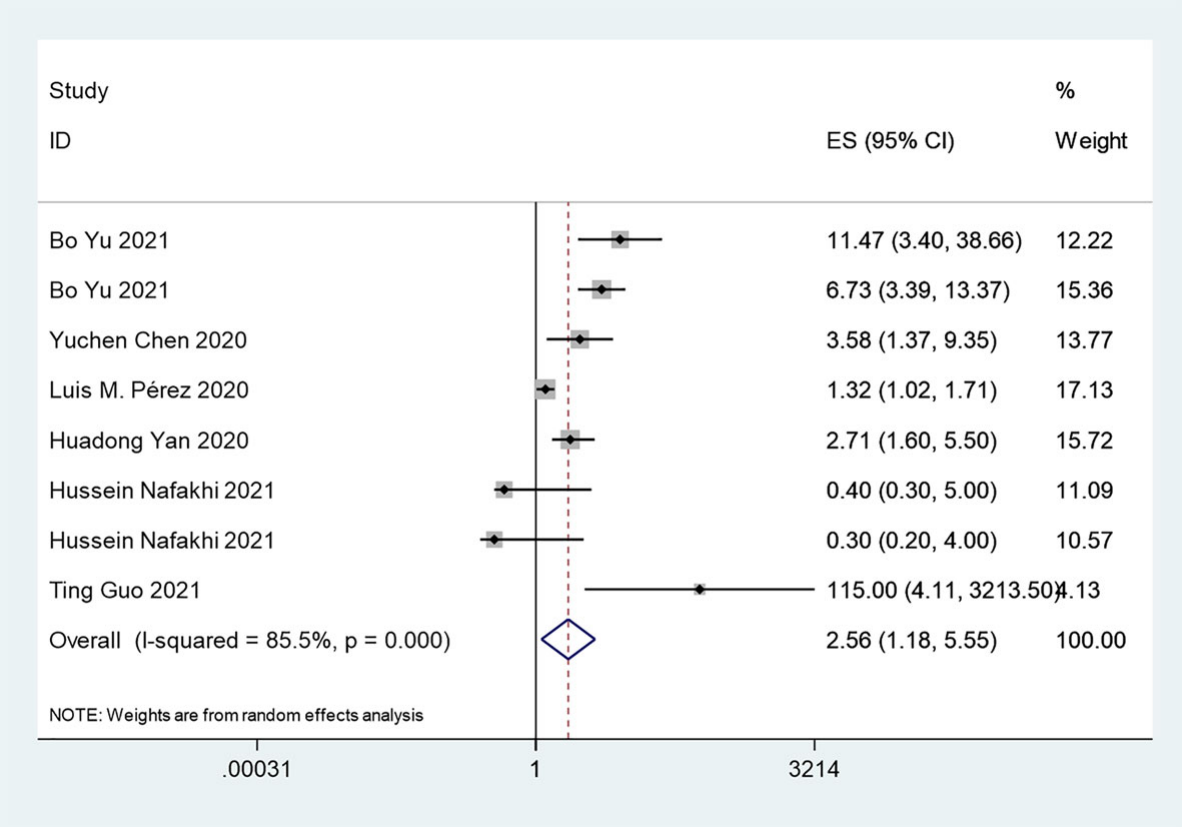
Forest plots of OR for the association between the insulin treatment and the complications of COVID-19.

The overall analysis included 8 studies that reported quantitative data regarding severe/critical COVID-19 illness. All extracted data were divided according to the severe/critical illness status based on the standard illness severity level (12). The overall risk of severe/critical COVID-19 illness was higher in the insulin treatment group than the control group (OR = 2.56, 95% CI, 1.18–5.55, I^2^ = 85.5%, p<0.001) (**Figure 5**). Begg’s test (p = 0.902), Egger’s test (p = 0.308) and the symmetric distribution funnel plots (**Figure 3B**) indicated that publication bias did not exist. The results of the sensitivity analysis conducted by excluding one study at a time did not change (**Figure 4B**).

#### Insulin Treatment and In-Hospital Admission for COVID-19

**Figure 6:**
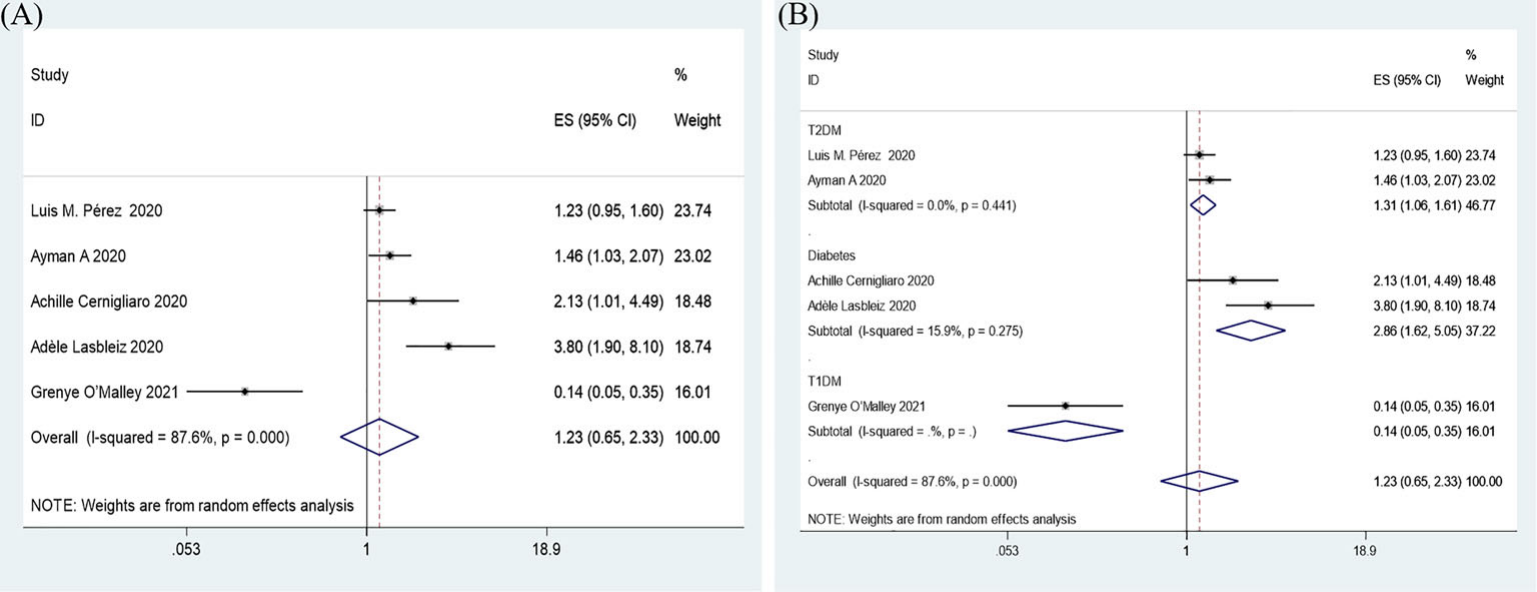
Forest plots of OR for the overall result **(A)** and subgroup analysis based on type of diabetes **(B)** of the association between the insulin treatment and the in-hospital admission of COVID-19.

Overall, insulin therapy was not associated with in-hospital admission for COVID-19 (OR=1.23; 95% CI, 0.65-2.33) (**Figure 6A**). Strong heterogeneity existed among the 5 included articles (I^2^ statistic=87.6%, p<0.001). Interestingly, the subgroup based on the type of diabetes significantly decreased the heterogeneity in each subgroup (**Figure 6B**). In the T2DM and Diabetes subgroups (patients with diabetes of which type was not clear in the extracted study), insulin treatment significantly increased in-hospital admission for COVID-19 (T2DM, OR=1.31; 95% CI, 1.06-1.61; Diabetes, OR=2.86; 95% CI, 1.62-5.05), which was the opposite of the results in the T1DM subgroup (OR=0.14; 95% CI, 0.05-0.35). However, due to the limited number of included T1DM studies, the effects of insulin treatment on patients with COVID-19 and T1DM require further solid evidence. According to the results of the funnel analysis (**Figure 3C**), Begg’s test (p = 1.000), and Egger’s test (p = 0.861), publication bias could be excluded. The sensitivity analysis also indicated the stability of our results (**Figure 4C**).

#### Insulin Treatment and COVID-19 Hospitalization Time

**Figure 7:**
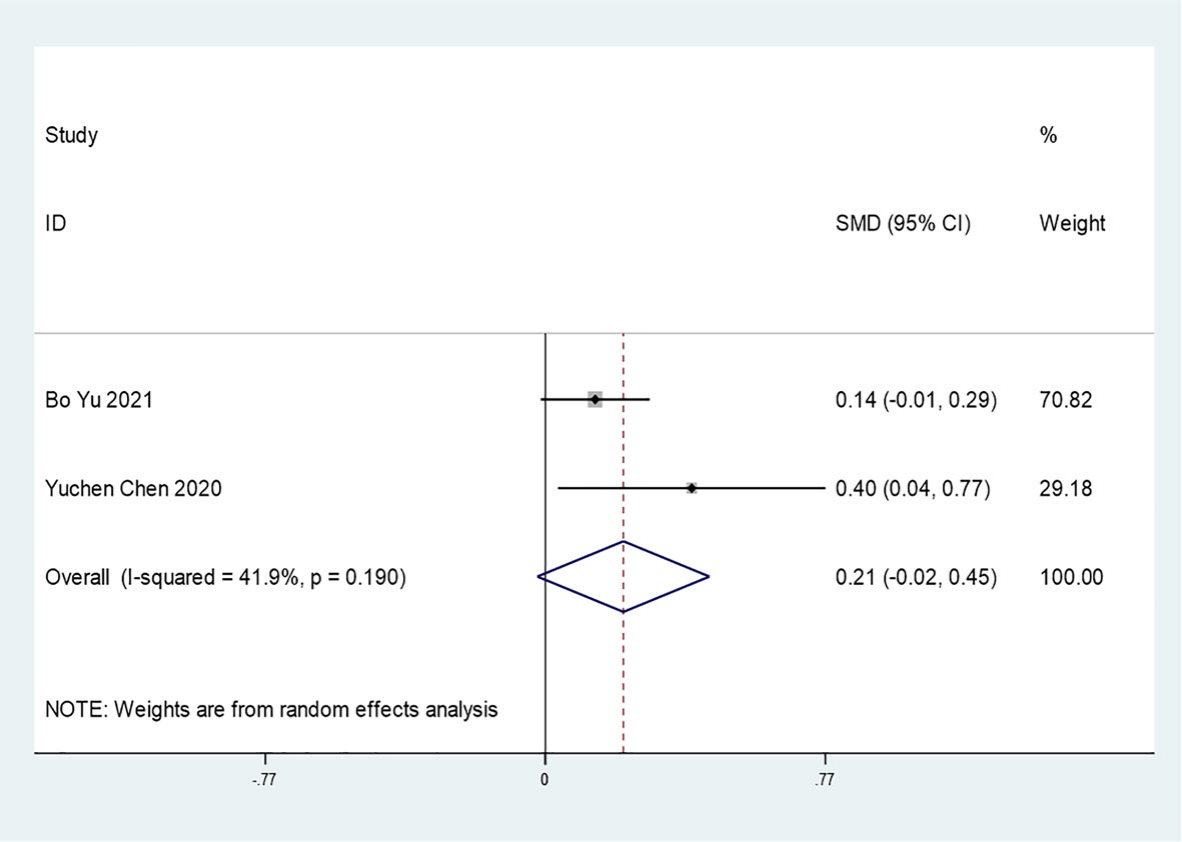
Forest plots of SMD for the association between the insulin treatment and the hospitalization time of COVID-19.

Only 2 studies mentioned the association between insulin treatment and COVID-19 hospitalization time (SMD=0.21 95% CI, -0.02-0.45). In general, insulin use did not affect the hospitalization time of COVID-19 patients (**Figure 7**). However, additional studies are needed to support this conclusion. Due to the limited number of included studies, further funnel plots or sensitivity analyses were not conducted.

### Secondary Outcomes

The overall data showed that insulin treatment led to increased adverse COVID-19 outcomes, including higher mortality, with moderate heterogeneity (I^2 =^ 69.2%, p<0.001) (**Figure 2**). To eliminate heterogeneity, further prespecified subgroup analyses based on the sample size, period of insulin treatment, type of diabetes and type of control group, were conducted (**Tables 2**, **3**). However, moderate/high heterogeneity remained in all subgroups (**Figures S1A–D**).

High heterogeneity also existed in the analysis of the effect of insulin treatment on severe/critical complications (I^2 =^ 85.5%, p<0.001) (**Figure 5**). We conducted further subgroup analyses based on the sample size (**Figure S2A**), period of insulin treatment (**Figure S2B**), and type of diabetes (**Figure S2C**), but moderate/high heterogeneity remained in all subgroups. Additionally, in the subgroup analysis based on the type of control group, heterogeneity was also present in the antidiabetic drug group (I^2 =^ 74.3% p=0.002), while there was decreased heterogeneity in the with and without antidiabetic drug groups (I^2 =^ 12.9% p=0.317) (**Figure S2D**). Overall, this meta-analysis still showed moderate/high heterogeneity after the subgroup analysis, indicating that the included articles failed to provide reliable information for further analysis.

## Discussion

Diabetes has been demonstrated to be among the most common comorbidities and is significantly associated with the mortality and severity of COVID-19 (16). Glucose-lowering medications have been proposed as a treatment for COVID-19. In contrast to preconception, insulin treatment has been demonstrated to be related to increased mortality and ICU admission for COVID-19 (14). In the current study, insulin treatment was associated with increased mortality and incidence of severe/critical complications in patients with COVID-19 and diabetes. Our study may provide evidence of the adverse effect of insulin treatment among patients with COVID-19 and diabetes, especially among those with type 2 diabetes (T2DM), as the subjects in most included studies suffered from T2DM. However, considering the limited number of studies concerning type 1 diabetes (T1DM) in our meta-analysis, the association between insulin treatment and adverse outcomes in patients with COVID-19 and T1DM are needed to be investigated in more large-scale clinical studies.

### Association Between Insulin Treatment and COVID-19

Our meta-analysis mainly indicates that insulin treatment may be associated with increased mortality (**Figure 2**) and severe/critical complications in T2DM and COVID-19 patients (**Figure 5**). Insulin treatment may also increase in-hospital admission of patients with T2DM and COVID-19 (**Figure 6B**), while there may be no significant relationship between insulin treatment and hospitalization time (**Figure 7**). The patients in the included studies of this meta-analysis had used insulin before catching COVID 19, so the results in our meta-analysis should be interpreted with caution as insulin is usually given to patients in a late stage of diabetes, since it is difficult to rule out the negative effect of advanced diabetes on the poor outcome.

### Mechanism Underlying the Relationship Between Insulin Treatment and Worse COVID-19 Outcomes

Although the mechanism underlying the association between insulin use and the poor outcome of COVID-19 remains unclear, there are several possible explanations for these results. First of all, diabetes itself contributed to a low grade of chronic inflammation, which characterized by impaired innate and adaptive inflammation system. This disorder of inflammation would lead to the dysregulation of the immune response with higher level of proinflammatory factors and lower anti-inflammatory cytokines. Insulin treatment was demonstrated to increase macrophage-produced inflammatory cytokine levels in the context of lipopolysaccharide-induced sepsis (31). Additionally, insulin was associated with increased lung inflammation in a sepsis model of diabetic rats (32), while lung cells are the main locations for COVID-19 inflammation. Therefore, we speculate that insulin treatment may participate in the development of COVID-19 by promoting proinflammatory system to aggravate inflammation disorder and pulmonary disease.

Moreover, insulin, an anabolic hormone to improve energy storage, is to increase appetite and weight gain, which contributed to obesity with inhibited lipolysis in adipose and muscle tissues (33, 34). Obesity people is characterized by chronic inflammation and impaired innate immunity with ample production of proinflammatory factors including tumor necrosis factor (TNF-α) and interleukin-6 (IL-6), which also comprised one of risk factors for COVID-19. Consistent with this, obesity and its impaired inflammatory imbalance contributed by insulin treatment may also be one of the mechanisms underlying the relationship between insulin use and COVID-19 outcomes.

Furthermore, hypoglycemia is among the most common adverse effects of insulin treatment, especially in the context of intensive care (35, 36). Insulin treatment increased the incidence of hypoglycemia in patients with COVID-19 and type 2 diabetes mellitus (T2DM) (14), which may impair the glucose homeostasis and glucose control to affect the COVID-19 outcomes. However, there must be other unknown mechanisms underlying the effect of insulin treatment on the outcomes of COVID-19, as the effect still remains in patients without hypoglycemia during hospitalization.

Although the potential mechanisms of the deleterious effect of insulin treatment on COVID-19 progress are discussed above, considering the irreplaceable role of insulin in controlling glucose and diabetes complications, it may be not advisable to give up insulin therapy before finding better or safer hypoglycemic agents for COVID-19 and T2DM patients. Moreover, Critical unwell patients with COVID-19 and diabetes have high insulin requirements and poorer time in optimal target range for blood glucose during peak inflammatory response (37), which indicating the insulin treatment is necessary during the late stage of COVID-19 disease. Additionally, the effects of insulin usage in COVID-19 progression are also affected by the blood glucose level and severity of diabetes (16, 17). Therefore, the results from the current meta-analysis should be interpreted with caution as the complicated situation during different stages of diabetes and insulin effectively in controlling glycemic levels, and more convinced well-designed randomized controlled trials are required to provide more precise conclusions for insulin usage in COVID-19 progression.

### Theoretical and Practical Significance

Our study is to summarize the effects of insulin therapy and clinical in outcomes of patients with COVID-19 and diabetes. Additionally, we raised an interesting and significant question regarding the mechanism underlying the relationship between insulin and the development of COVID-19, as there are no reasonable explanations. Therefore, the timing or dose of insulin treatment in patients with COVID-19 and diabetes may be an urgent public problem that must be addressed to control the development of COVID-19.

### Limitations of the Study

There are several limitations in our study. First, the high heterogeneity in the results cannot be ignored, and most of the study included was observational study, many biases could not be controlled. Second, the type of diabetes was unclear in several included studies, potentially rendering our conclusion not generalizable to all types of diabetes. Notably, one report focusing on the protective effect of insulin treatment on COVID-19 outcomes involved patients with T1DM and, revealed the opposite conclusion from our results (including mostly T2DM patients), indicating that a different approach to insulin therapy may be needed according to the type of diabetes. Therefore, our study is more likely to be applicable to T2DM patients, while guidance for insulin treatment in COVID-19 patients with different types of diabetes, especially T1DM, needs more clinical research. Third, the causal relationship between insulin treatment and COVID-19 outcomes remains vague due to the limited amount of prospective data in our included studies. Moreover, this systematic review and meta-analysis was not registered in PROSPERO, representing another limitation of our study. Last but not least, the results from this study are only based on observational studies which have many confounders, therefore the evidence generated from this study is not strong enough. More randomized clinical trials are still needed to confirm the results from this study.

### Conclusion

In summary, our study revealed that insulin treatment is associated with increased mortality and other severe/critical complications of COVID-19. Our findings could provide clinical guidance for insulin treatment in patients with COVID-19 and diabetes. Further randomized controlled trial was needed to confirm this finding.

## Data Availability Statement

The original contributions presented in the study are included in the article/**Supplementary Material**. Further inquiries can be directed to the corresponding authors.

## Ethics Statement

Ethical review and approval was not required for the study on human participants in accordance with the local legislation and institutional requirements. Written informed consent for participation was not required for this study in accordance with the national legislation and the institutional requirements.

## Author Contributions

Conceptualization, YY. Methodology, ZC, JZ and YY. Software, YY. Validation, JZ, YY and ZC. Formal analysis, YY and ZC. Investigation, YY. Resources, JZ. Data curation, ZC. Writing—original draft preparation, YY. Writing—review and editing, JZ. Visualization, JZ. Supervision, ZC and JZ. Project administration, JZ. Funding acquisition, JZ. All authors contributed to the article and approved the submitted version.

## Funding

This work was supported by grants from the National Natural Science Foundation of China [82070807, 91749118, 81770775, 81730022], the Planned Science and Technology Project of Hunan Province [2017RS3015] and National key research and development program [2019YFA0801903, 2018YFC2000100].

## Conflict of Interest

The authors declare that the research was conducted in the absence of any commercial or financial relationships that could be construed as a potential conflict of interest.

## Supplementary Material

Supplementary Figure 1 Outcomes of the subgroup analysis of the sample size **(A)**, period of insulin treatment **(B)**, type of diabetes **(C)**, kind of control groups **(D)** of the insulin treatment with mortality in patients with T2DM by forest plots.

Supplementary Figure 2 Outcomes of the subgroup analysis of the sample size **(A)**, period of insulin treatment **(B)**, type of diabetes **(C)**, kind of control groups **(D)** of the insulin treatment with sever/critical complications in patients with T2DM by forest plots.

Supplementary Figure 3 Outcomes of the subgroup analysis of the period of insulin treatment **(A)**, kind of control groups **(B)** of the insulin treatment with the in-hospital admission by forest plots.

## References

[1] Bhadade R Harde M deSouza R Kasbe A Deshpande C Dave S. Appraisal of Critically Ill COVID-19 Patients at a Dedicated COVID Hospital The Effects of Type 2 Diabetes Mellitus on Organ Metabolism and the Immune System. J Assoc Phys India (2020) 68(9):14–9. 10.1016/s2213-8587(20)30271-0 32798339

[2] Abu-Farha M Al-Mulla F Thanaraj TA Kavalakatt S Ali H Abdul Ghani M. Impact of Diabetes in Patients Diagnosed With COVID-19. Front Immunol (2020) 11:576818. 10.3389/fimmu.2020.576818 33335527 PMC7736089

[3] Zhu L She ZG Cheng X Qin JJ Zhang XJ Cai J. Association of Blood Glucose Control and Outcomes in Patients With COVID-19 and Pre-Existing Type 2 Diabetes. Cell Metab (2020) 31(6):1068–77.e1063. 10.1016/j.cmet.2020.04.021 32369736 PMC7252168

[4] Wang S Ma P Zhang S Song S Wang Z Ma Y. Fasting Blood Glucose at Admission is an Independent Predictor for 28-Day Mortality in Patients With COVID-19 Without Previous Diagnosis of Diabetes: A Multi-Centre Retrospective Study. Diabetologia (2020) 63(10):2102–11. 10.1007/s00125-020-05209-1 PMC7347402 32647915

[5] Lee MH Wong C Ng CH Yuen DCW Lim AYL Khoo CM. Effects of Hyperglycaemia on Complications of COVID-19: A Meta-Analysis of Observational Studies. Diabetes Obes Metab (2021) 23(1):287–9. 10.1111/dom.14184 32869450

[6] Alahmad B Al-Shammari AA Bennakhi A Al-Mulla F Ali H. Fasting Blood Glucose and COVID-19 Severity: Nonlinearity Matters. Diabetes Care (2020) 43(12):3113–6. 10.2337/dc20-1941 PMC7770269 33051331

[7] Hariyanto TI Kurniawan A. Metformin Use Is Associated With Reduced Mortality Rate From Coronavirus Disease 2019 (COVID-19) Infection. Obes Med (2020) 19:100290. 10.1016/j.obmed.2020.100290 32844132 PMC7434427

[8] Hariyanto TI Kurniawan A. Dipeptidyl Peptidase 4 (DPP4) Inhibitor and Outcome From Coronavirus Disease 2019 (COVID-19) in Diabetic Patients: A Systematic Review, Meta-Analysis, and Meta-Regression. J Diabetes Metab Disord (2021) 20(1):1–8. 10.1007/s40200-021-00777-4 33816358 PMC8003892

[9] Riahi S Sombra LRS Lo KB Chacko SR Neto AGM Azmaiparashvili Z. Insulin Use, Diabetes Control, and Outcomes in Patients With COVID-19. Endocr Res (2020) 46(2):1–6. 10.1080/07435800.2020.1856865 33275067

[10] Pérez-Belmonte LM Torres-Peña JD López-Carmona MD Ayala-Gutiérrez MM Fuentes-Jiménez F Huerta LJ. Mortality and Other Adverse Outcomes in Patients With Type 2 Diabetes Mellitus Admitted for COVID-19 in Association With Glucose-Lowering Drugs: A Nationwide Cohort Study. BMC Med (2020) 18(1):359. 10.1186/s12916-020-01832-2 33190637 PMC7666969

[11] Moher D Liberati A Tetzlaff J Altman DG. Preferred Reporting Items for Systematic Reviews and Meta-Analyses: The PRISMA Statement. PLoS Med (2009) 6(7):e1000097. 10.1371/journal.pmed.1000097 19621072 PMC2707599

[12] Umpierrez G Rushakoff R Seley JJ Zhang JY Shang T Han J. Hospital Diabetes Meeting 2020a Territory-Wide Study on the Impact of COVID-19 on Diabetes-Related Acute Care. J Diabetes Sci Technol (2020) 14(5):928–44. 10.1136/bcr-2020-23672010.1177/1932296820939626 PMC7477766 32783456

[13] Stang A. Critical Evaluation of the Newcastle-Ottawa Scale for the Assessment of the Quality of Nonrandomized Studies in Meta-Analyses. Eur J Epidemiol (2010) 25(9):603–5. 10.1007/s10654-010-9491-z 20652370

[14] Yu B Li C Sun Y Wang DW. Insulin Treatment Is Associated With Increased Mortality in Patients With COVID-19 and Type 2 Diabetes. Cell Metab (2021) 33(1):65–77.e62. 10.1016/j.cmet.2020.11.014 33248471 PMC7682421

[15] Cariou B Hadjadj S Wargny M Pichelin M Al-Salameh A Allix I. Phenotypic Characteristics and Prognosis of Inpatients With COVID-19 and Diabetes: The CORONADO Study. Diabetologia (2020) 63(8):1500–15. 10.1007/s00125-020-05180-x PMC7256180 32472191

[16] Chen Y Yang D Cheng B Chen J Peng A Yang C. Clinical Characteristics and Outcomes of Patients With Diabetes and COVID-19 in Association With Glucose-Lowering Medication. Diabetes Care (2020) 43(7):1399–407. 10.2337/dc20-0660 32409498

[17] Sardu C D’Onofrio N Balestrieri ML Barbieri M Rizzo MR Messina V. Outcomes in Patients With Hyperglycemia Affected by COVID-19: Can We Do More on Glycemic Control? Diabetes Care (2020) 43(7):1408–15. 10.2337/dc20-0723 PMC7305003 32430456

[18] Agarwal S Schechter C Southern W Crandall JP Tomer Y. Preadmission Diabetes-Specific Risk Factors for Mortality in Hospitalized Patients With Diabetes and Coronavirus Disease 2019. Diabetes Care (2020) 43(10):2339–44. 10.2337/dc20-1543 PMC7510015 32769128

[19] Shestakova MV Vikulova OK Isakov M Dedov II. [Diabetes and COVID-19: Analysis of the Clinical Outcomes According to the Data of the Russian Diabetes Registry]. Problemy Endokrinol (2020) 66(1):35–46. 10.14341/probl12458 33351311

[20] Mirani M Favacchio G Carrone F Betella N Biamonte E Morenghi E. Impact of Comorbidities and Glycemia at Admission and Dipeptidyl Peptidase 4 Inhibitors in Patients With Type 2 Diabetes With COVID-19: A Case Series From an Academic Hospital in Lombardy, Italy. Diabetes Care (2020) 43(12):3042–9. 10.2337/dc20-1340 33023989

[21] Khalili S Moradi O Kharazmi AB Raoufi M Sistanizad M Shariat M. Comparison of Mortality Rate and Severity of Pulmonary Involvement in Coronavirus Disease-2019 Adults Patients With and Without Type 2 Diabetes: A Cohort Study. Can J Diabetes (2020) S1499-2671(20):30426–3. 10.1016/j.jcjd.2020.10.014 PMC7604035 33339741

[22] Turcotte JJ Meisenberg BR MacDonald JH Menon N Fowler MB West M. Risk Factors for Severe Illness in Hospitalized Covid-19 Patients at a Regional Hospital. PLoS One (2020) 15(8):e0237558. 10.1371/journal.pone.0237558 32785285 PMC7423129

[23] Yan H Valdes AM Vijay A Wang S Liang L Yang S. Role of Drugs Used for Chronic Disease Management on Susceptibility and Severity of COVID-19: A Large Case-Control Study. Clin Pharmacol Ther (2020) 108(6):1185–94. 10.1002/cpt.2047 32910830

[24] Al Hayek AA Robert AA Matar AB Algarni A Alkubedan H Alharbi T. Risk Factors for Hospital Admission Among COVID-19 Patients With Diabetes. A Study Saudi Arabia Saudi Med J (2020) 41(10):1090–7. 10.15537/smj.2020.10.25419 PMC7841525 33026050

[25] Cernigliaro A Allotta AV Scondotto S. [Can Diabetes and its Related Hypoglycemic Drug Treatment be Considered Risk Factors for Health Outcomes in COVID-19 Patients? The Results of a Study in the Population Residing in Sicily Region (Southern Italy)]. Epidemiol e Prevenzione (2020) 44(5-6 Suppl 2):315–22. 10.19191/ep20.5-6.S2.132 33412824

[26] Nafakhi H Alareedh M Al-Buthabhak K Shaghee F Nafakhi A Kasim S. Predictors of Adverse in-Hospital Outcome and Recovery in Patients With Diabetes Mellitus and COVID-19 Pneumonia in Iraq. Diabetes Metab Syndr (2021) 15(1):33–8. 10.1016/j.dsx.2020.12.014 PMC7832757 33296788

[27] Lasbleiz A Cariou B Darmon P Soghomonian A Ancel P Boullu S. Phenotypic Characteristics and Development of a Hospitalization Prediction Risk Score for Outpatients With Diabetes and COVID-19: The DIABCOVID Study. J Clin Med (2020) 9(11):3726. 10.3390/jcm9113726 PMC7699790 33233575

[28] O’Malley G Ebekozien O Desimone M Pinnaro CT Roberts A Polsky S. COVID-19 Hospitalization in Adults With Type 1 Diabetes: Results From the T1D Exchange Multicenter Surveillance Study. J Clin Endocrinol Metab (2021) 106(2):e936–42. 10.1210/clinem/dgaa825 PMC7717244 33165563

[29] Guo T Shen Q Ouyang X Guo W Li J He W. Clinical Findings in Diabetes Mellitus Patients With COVID-19. J Diabetes Res (2021) 2021:7830136. 10.1155/2021/7830136 33506052 PMC7811569

[30] Lally MA Tsoukas P Halladay CW O’Neill E Gravenstein S Rudolph JL. Metformin is Associated With Decreased 30-Day Mortality Among Nursing Home Residents Infected With SARS-Cov2. J Am Med Directors Assoc (2021) 22(1):193–8. 10.1016/j.jamda.2020.10.031 PMC7586924 33232684

[31] Brundage SI Kirilcuk NN Lam JC Spain DA Zautke NA. Insulin Increases the Release of Proinflammatory Mediators. J Trauma (2008) 65(2):367–72. 10.1097/TA.0b013e3181801cc0 18695473

[32] Filgueiras LR Capelozzi VL Martins JO Jancar S. Sepsis-Induced Lung Inflammation is Modulated by Insulin. BMC Pulm Med (2014) 14:177. 10.1186/1471-2466-14-177 25398720 PMC4251940

[33] Jacob S Hauer B Becker R Artzner S Grauer P Löblein K. Lipolysis in Skeletal Muscle is Rapidly Regulated by Low Physiological Doses of Insulin. Diabetologia (1999) 42(10):1171–4. 10.1007/s001250051288 10525656

[34] Kolb H Kempf K Röhling M Martin S. Insulin: Too Much of a Good Thing Is Bad. BMC Med (2020) 18(1):224. 10.1186/s12916-020-01688-6 32819363 PMC7441661

[35] Gerstein HC Miller ME Byington RP Goff DC Jr. Bigger JT Buse JB. Effects of Intensive Glucose Lowering in Type 2 Diabetes. N Engl J Med (2008) 358(24):2545–59. 10.1056/NEJMoa0802743 PMC4551392 18539917

[36] Hypoglycemia in the Diabetes Control and Complications Trial The Diabetes Control and Complications Trial Research Group. Diabetes (1997) 46(2):271–86. 10.2337/diabetes.46.2.271 9000705

[37] Wu L Girgis CM Cheung NW. COVID-19 and Diabetes: Insulin Requirements Parallel Illness Severity in Critically Unwell Patients. Clin Endocrinol (Oxf) (2020) 93(4):390–3. 10.1111/cen.14288 PMC7404426 32683745

