# Supplementary figures and images for: Insulin Treatment May Increase Adverse Outcomes in Patients With COVID-19 and Diabetes: A Systematic Review and Meta-Analysis
Source: Front Endocrinol (Lausanne). 2021 Jul 22;12:696087. doi: 10.3389/fendo.2021.696087 (PMC8339900; doi:10.3389/fendo.2021.696087)

## Slide 1
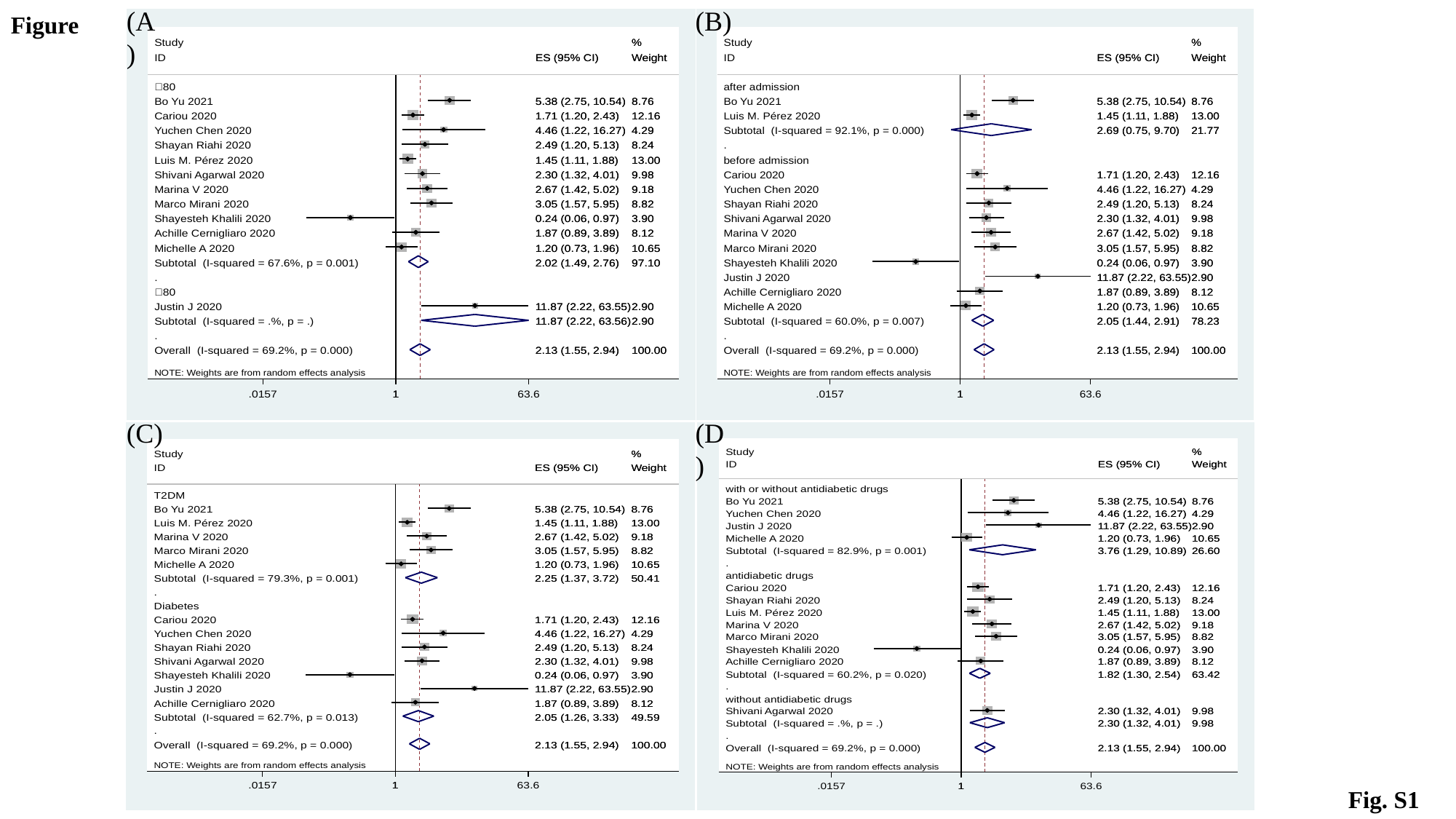

(A)
(B)
(C)
(D)
Figure
Fig. S1

## Slide 2
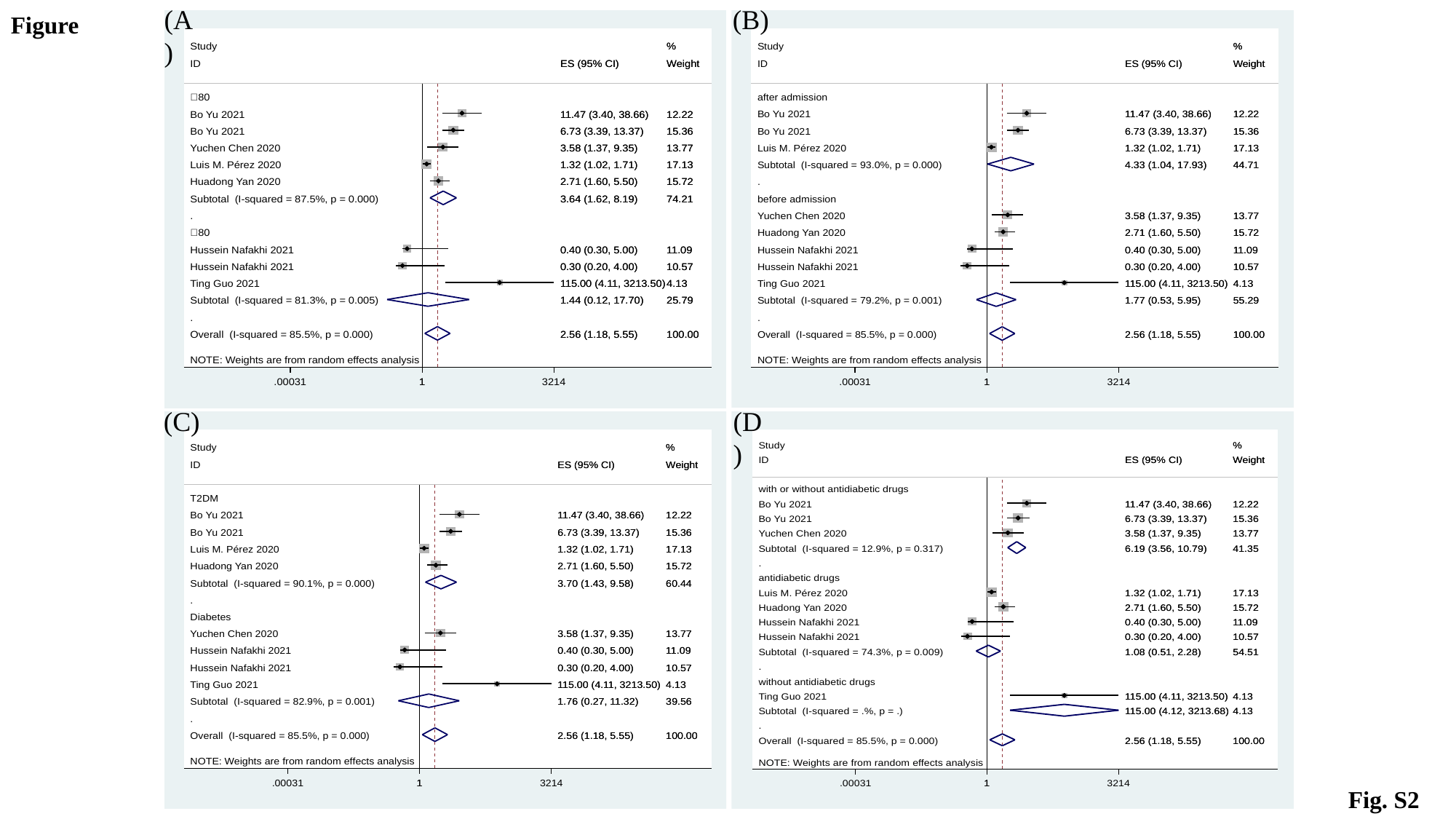

(A)
(B)
(C)
(D)
Figure
Fig. S2

## Slide 3
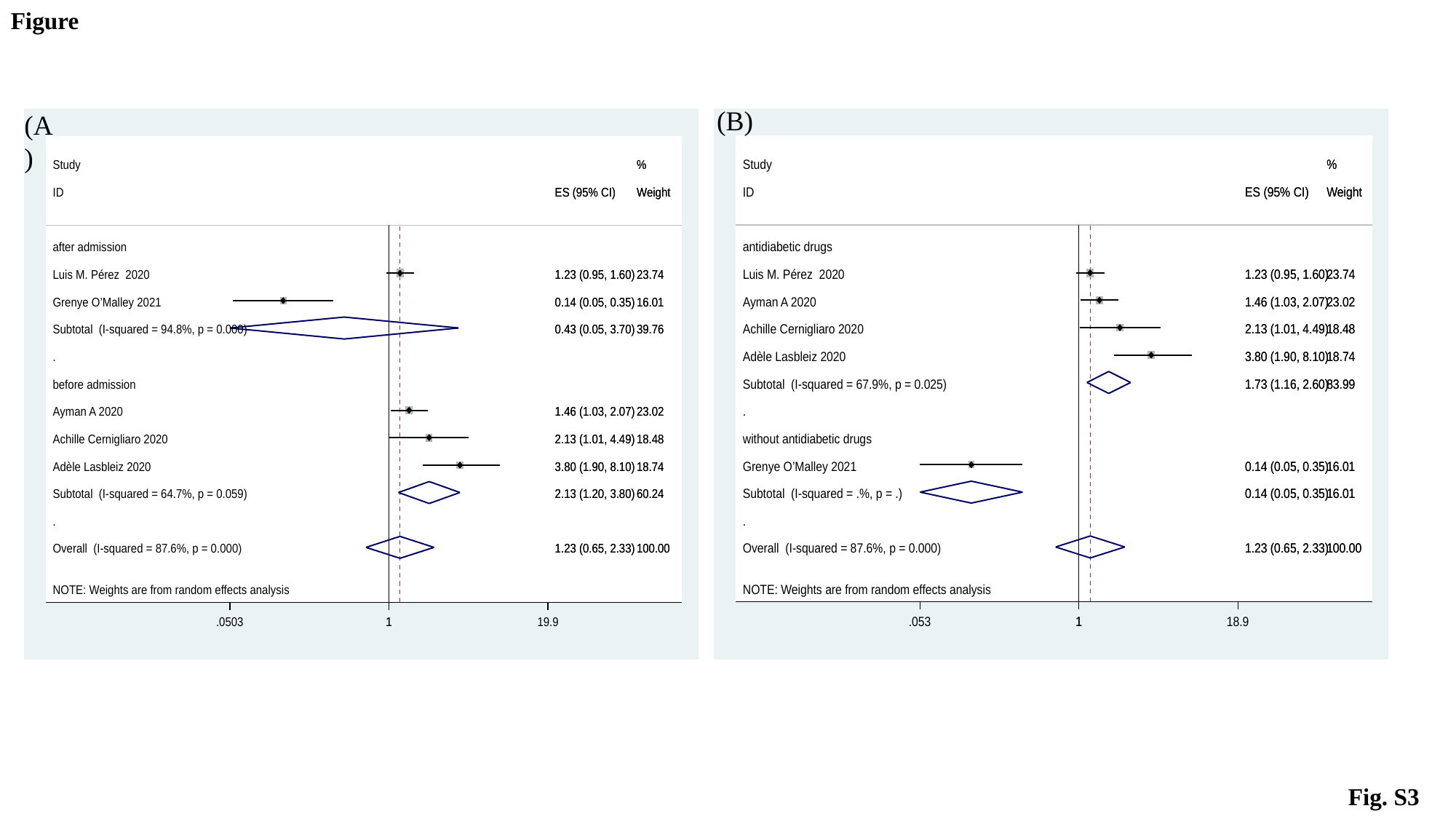

Figure
(B)
(A)
Fig. S3

Supplement: Supplementary Figure 1 — Outcomes of the subgroup analysis of the sample size (A), period of insulin treatment (B), type of diabetes (C), kind of control groups (D) of the insulin treatment with mortality in patients with T2DM by forest plots. [file Presentation_1.pptx]
